# Supplementary material for: Loss of vascular endothelial notch signaling promotes spontaneous formation of tertiary lymphoid structures
Source: Nat Commun. 2022 Apr 19;13:2022. doi: 10.1038/s41467-022-29701-x (PMC9018798; doi:10.1038/s41467-022-29701-x)
Supplement: Supplementary file 1 — Supplementary Information [file 41467_2022_29701_MOESM1_ESM.pdf]

Supplementary Material

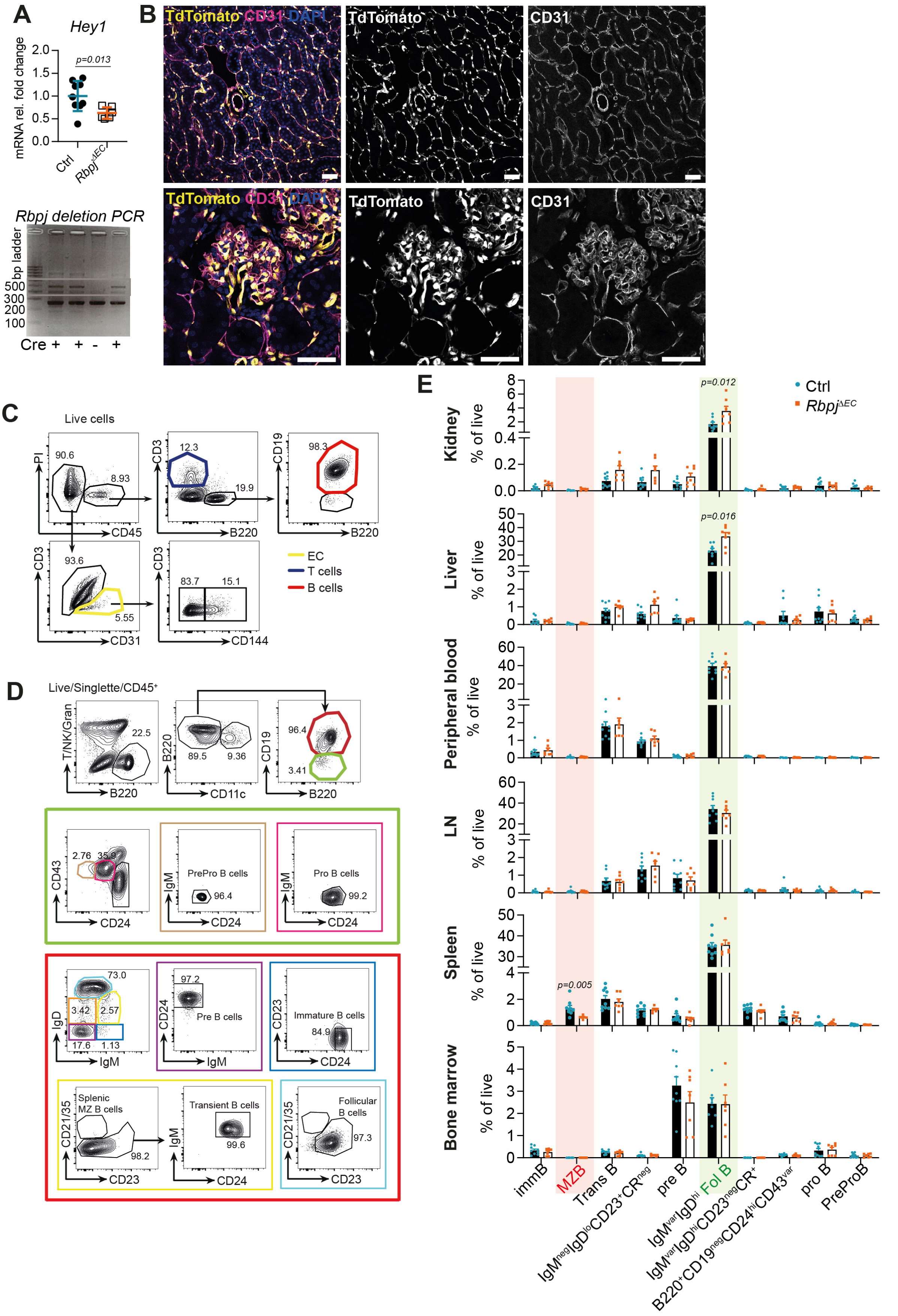

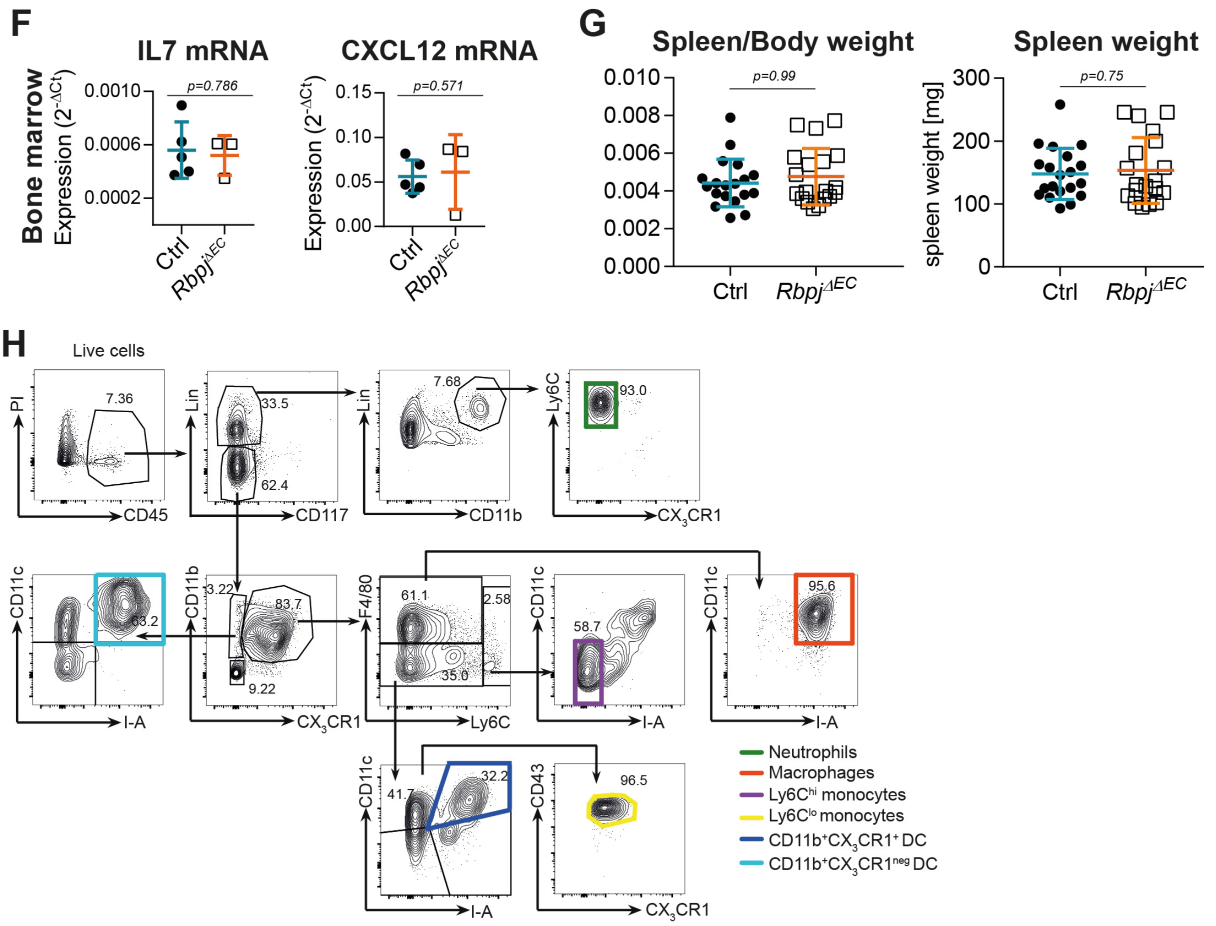

Cell Type	Phenotype
B cells	CD45+CD3negB220+CD19+
Follicular B cells	CD45+Lin(CD3/Ly6G/NK1.1)neg B220+CD19+IgDhiIgMlo/negCD21/35negCD23+
T cells	CD45+B220negCD19negCD3+
CD4+ T cells	CD45+B220negNK1.1negCD3+CD4+CD8aneg
CD8+ T cells	CD45+B220negNK1.1negCD3+CD4negCD8a+
NK cells	CD45+B220negNK1.1+CD3neg
iNKT cells	CD45+B220negNK1.1+CD3+
Neutrophils	CD45+Lin(CD19/CD3/Ly6G/NK1.1)+ CD11b+ CX3CR1negLy6Cint
Ly6Chi (inflammatory monoc.)	CD45+Lin(CD19/CD3/Ly6G/NK1.1)negCD11b+ CX3CR1+Ly6Chi F4/80lo/neg
Ly6Clo (patrolling monocytes)	CD45+Lin(CD19/CD3/Ly6G/NK1.1)negCD11b+ CX3CR1+Ly6Clow/negF4/80lo/negCD11cint I-AnegCD43+
Macrophages	CD45+Lin(CD19/CD3/Ly6G/NK1.1)negCD11b+ CX3CR1+Ly6Clow/negF4/80hiCD11c+I-A+
CD11b+CX3CR1+ Dendritic Cells	CD45+Lin(CD19/CD3/Ly6G/NK1.1)negCD11b+ CX3CR1+Ly6Clow/negF4/80lo/negCD11c+I-A+
CD11b+CX3CR1neg Dendritic Cells	CD45+Lin(CD19/CD3/Ly6G/NK1.1)negCD11b+ CX3CR1negCD11c+I-A+
Endothelial Cells	CD45negCD31+CD144var

Supplementary Figure 1. Analysis of renal targeting and cell populations after endothelial-specific loss of Notch signaling

A) Recombination control: upper panel, Whole kidney mRNA expression of *Hey1*, downstream of *Rbpj*, as internal control of endothelial *Rbpj* knockdown. CTRL n=9, $Rbpj^{\Delta EC}$ n=6, * p<0.05 (Mann-Whitney test, two-tailed, exact p=0.013) Graph: Scatter dot blot with mean and SD (whiskers).

Lower panel, left side, kidney PCR for Rbpj deletion band (at 500bp, only upon successful deletion of exons 6 and 7). **B**) $Cdh5Cre^{ERT2};TdTomato^{fl/fl}$ mouse model as recombination control: nuclear and cytoplasm TdTomato (yellow and middle column)

and CD31 staining (cytoplasmatic, purple and right column) show good recombination; exemplary image, kidney sections from n=3 mice stained. Scale bars, 50µm. **C**) Flow cytometry gating strategy for Endothelial cells, B and T lymphocytes. **D**) Flow cytometry gating strategy for B cell subset and progenitor panel. **E**) Flow cytometry of B cell subpopulations in kidney, liver, blood, lymph node, spleen and bone marrow. Follicular B cells (green shade) significantly upregulated in kidney (p=0.012) and liver (p=0.016). Marginal zone B cells (red shade) slightly lower in *Rbpj*$^{\Delta EC}$ spleen (p=0.005). No other significant change between groups observed. Box plots with mean, 25-75 percentile (bounds) and min/max (whiskers). Two-tailed Mann Whitney test. **F**) Bone marrow mRNA expression of IL7 (p=0.786) and CXCL12 (p=0.571) between groups; Mann-Whitney test, two-tailed; scatter dot blot with mean and SD (whiskers), CTRL n=5, *Rbpj*$^{\Delta EC}$ n=3. **G**) Spleen weight (p=0.99) and spleen/body weight ratio (p=0.75) in Ctrl and *Rbpj*$^{\Delta EC}$.; Scatter dot blots with mean, SD (whiskers); two-tailed Mann-Whitney test; CTRL n=20, *Rbpj*$^{\Delta EC}$ n=19. **H**) Flow cytometry gating strategy for Myeloid panel. Table, list of markers used for each cell type. Source data are provided with this paper.

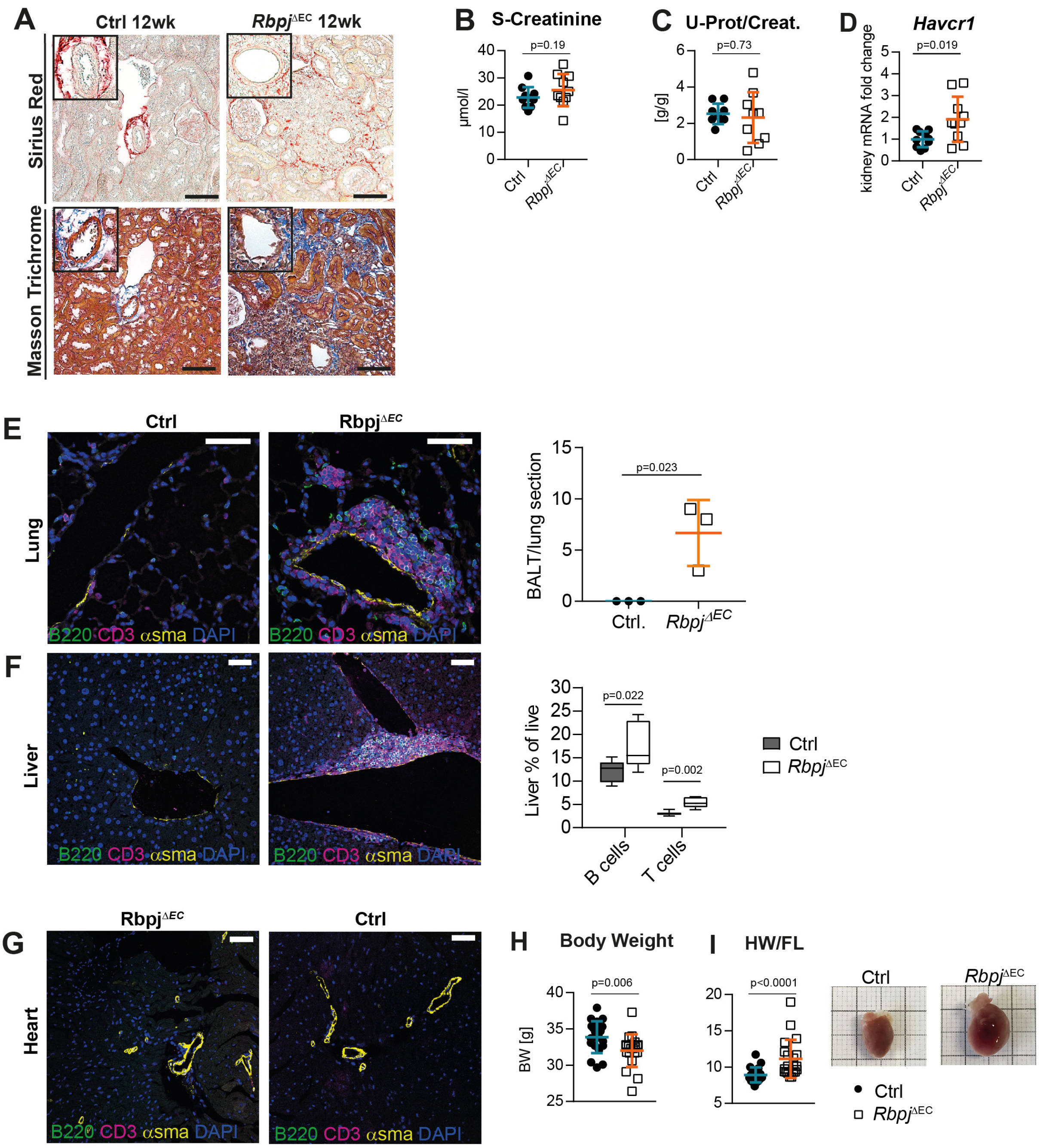

Supplementary Figure 2: Renal and cardiac parameters in Rbpj-mutant mice.

A) Sirius red (upper panel) and Masson trichrome (lower panel) staining of Ctrl (left) and *Rbpj*$^{\Delta EC}$ kidneys (right). Insets, arterial cross section. Scale bar, 50µm. Exemplary images, kidneys from n=5 mice/group were stained. **B**) Serum creatinine, n= 10 mice/group, scatter dot blot with mean, SD (whiskers), Mann-Whitney test, two-tailed, p=0.19, **C**) Urine protein to creatinine ratio between groups. n= 10 mice/group, scatter dot blot with mean, SD (whiskers), Mann-Whitney test, two-tailed, p=0.73 **D**) Whole kidney mRNA *Havcr1* (Kim1) expression, n= 10 mice per group from 2 independent experiments, scatter dot blot with mean, SD (whiskers), p=0.019 (Mann-Whitney test, two-tailed).

E) Immunofluorescence costaining of B220 (green), CD3 (purple) and αsma (yellow) of Ctrl and *Rbpj*$^{\Delta EC}$ lungs. Scale bars, 50µm. Quantification: average number of TLS counted per lung section, each data point represents one animal (n=3 mice per group), scatter dot blot with mean, SD (whiskers), Unpaired Student's t-test, two-sided, p=0.0229, *p<0.05. **F**) Immunofluorescence costaining of B220 (green), CD3 (purple) and αsma (yellow) of Ctrl and *Rbpj*$^{\Delta EC}$ liver. Scale bars, 50µm. Right, quantification via flow cytometry staining, % of live. N= 5 mice per group. Mean, Box from 25.-75. Percentile, whiskers represent min/max. B-cells, p=0.022; T-cells, p=0.002, Mann-Whitney Test, two-tailed.

G) Immunofluorescence costaining of B220 (green), CD3 (purple) and αsma (yellow) of Ctrl and *Rbpj*$^{\Delta EC}$ hearts, exemplary pictures, no quantification, representative of n=5/group. Scale bar = 50µm. **H**) Body weight (n=20/group, p=0.0062) and **I**) heart weight to femur length ration (HW/FL), (n=20/group, p<0.0001) in 20-22 weeks old mice (12 weeks after Tamoxifen injection). H&I: Mann-Whitney test, two-tailed. Graphs: Scatter dot blot with mean and SD (whiskers). Source data are provided with this paper.

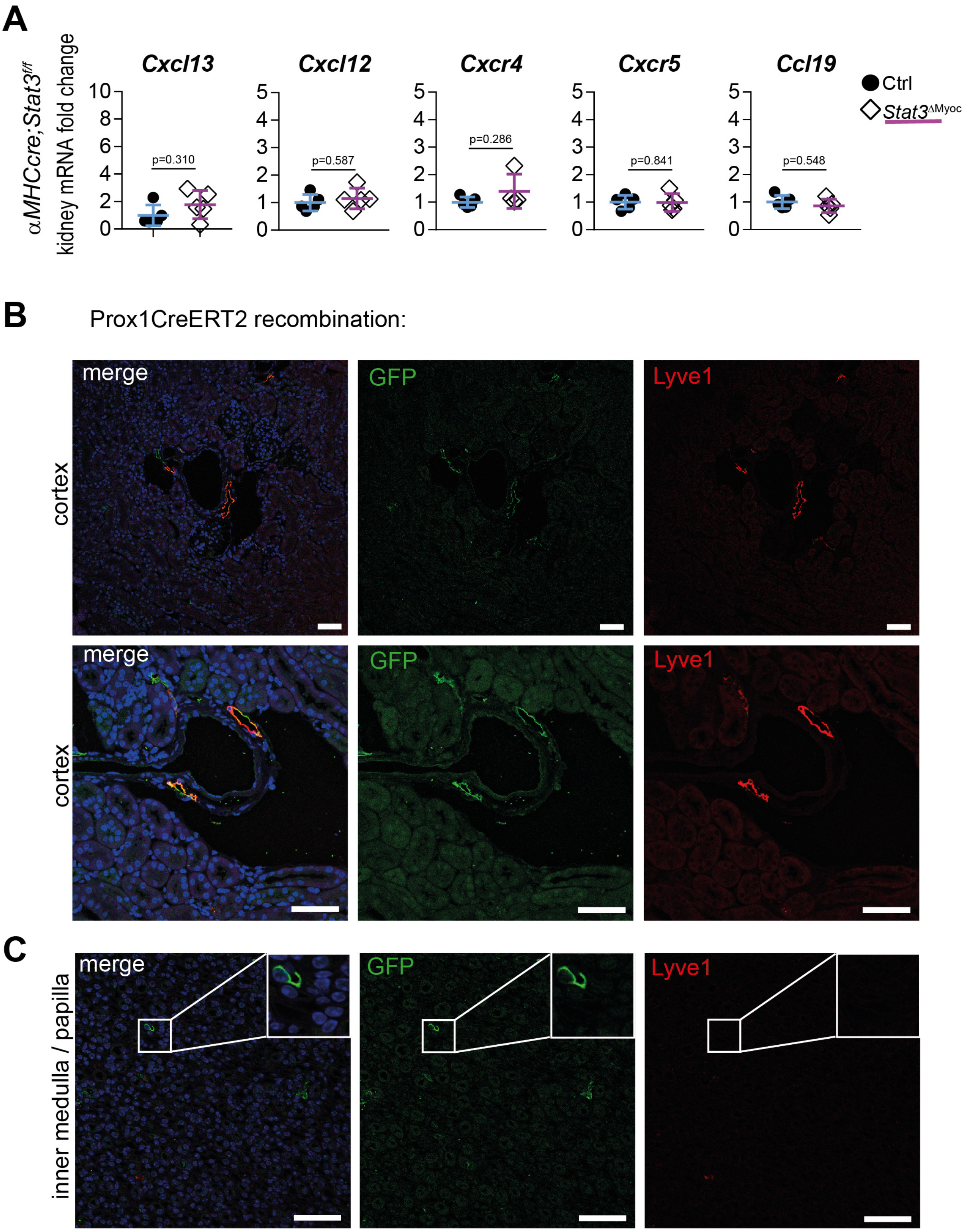

Supplementary Figure 3: Gene / transgene expression in Stat3 $^{\Delta Myoc}$ and *Prox1Cre*^{ERT2} recombination control kidneys

A) Kidney mRNA expression of Cxcl13 (p=0.310), Cxcl12 (p=587), Cxcr4 (p=0.286), Cxcr5 (p=0.841) and Ccl19 (p=0.548) in Stat3$^{\Delta Myoc}$ vs. Control. N=5/group. Scatter dot

plots with mean and SD (whiskers), two-tailed Mann Whitney test. Experiment repeated 2x with similar results. Source data are provided with this paper.

B and **C**: Recombination control of *Prox1Cre*^{ERT2} expression in adult mouse kidney (*Prox1Cre*^{ERT2}*;mTmG* mouse model). Prox1-GFP in green, Lyve1-costaining in red. Exemplary pictures, several sections on different levels of the kidney were stained from n=2 mice. **A**) cortex area; **B**) inner medulla/papilla area. All scale bars 50µm.

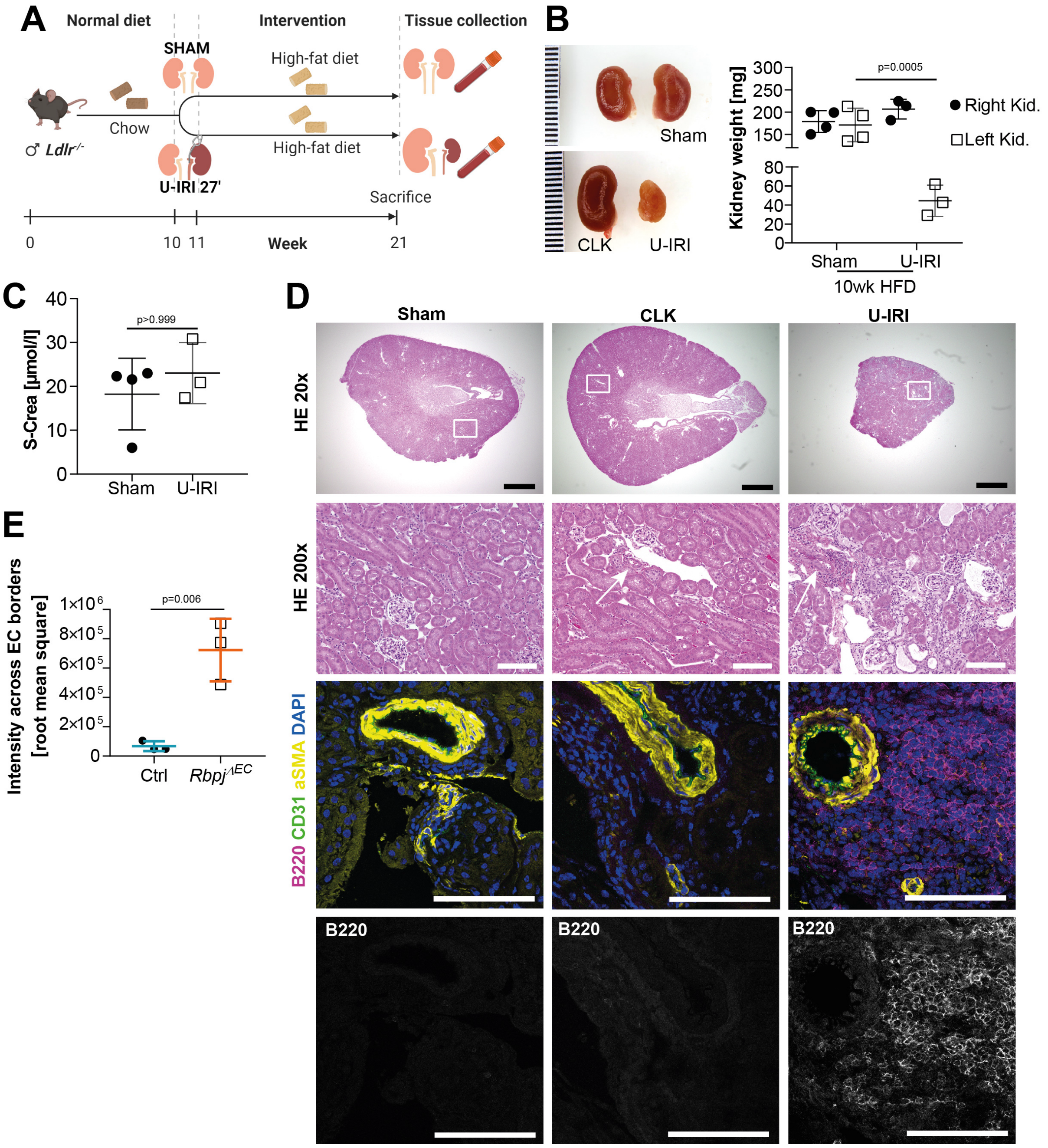

Supplementary Figure 4: Inflammatory kidney damage model using unilateral IRI and high fat diet. **A**) Sketch of experimental protocol. 4A-D, n=4 Ctrl, n=3 U-IRI. **B**) Kidneys of Sham and U-IRI mice, mm-scale on left. Right panel, kidney weight in mg. Scatter dot plot with mean and SD (whiskers), *** p=0.0005, Multiple T tests, Two-stage linear step-up procedure of Benjamini, Krieger and Yekutieli, with Q = 1%.**C**) serum creatinine, scatter dot blot, mean, SD (whiskers). P=0.99, two-tailed Mann Whitney test. **D**) HE (upper panels; arrow, arterial lumen) and B220 CD31 αsma immunofluorescence costaining (third panel, lowest panel B220 channel only) in

Sham, contralateral kidney and U-IRI kidney. Representative images of n=4 Sham mice and n=3 CLK/U-IRI kidneys. Scale bars: Black bar, 1mm; white bars 100μm.

E) Referring to figure 4: Quantification of PNAd staining on kidney endothelium; root mean square of greyscale intensity values across EC borders. Scatter dot plot with mean and SD (whiskers). n=3 mice/group,p=0.0063. Source data are provided with this paper.

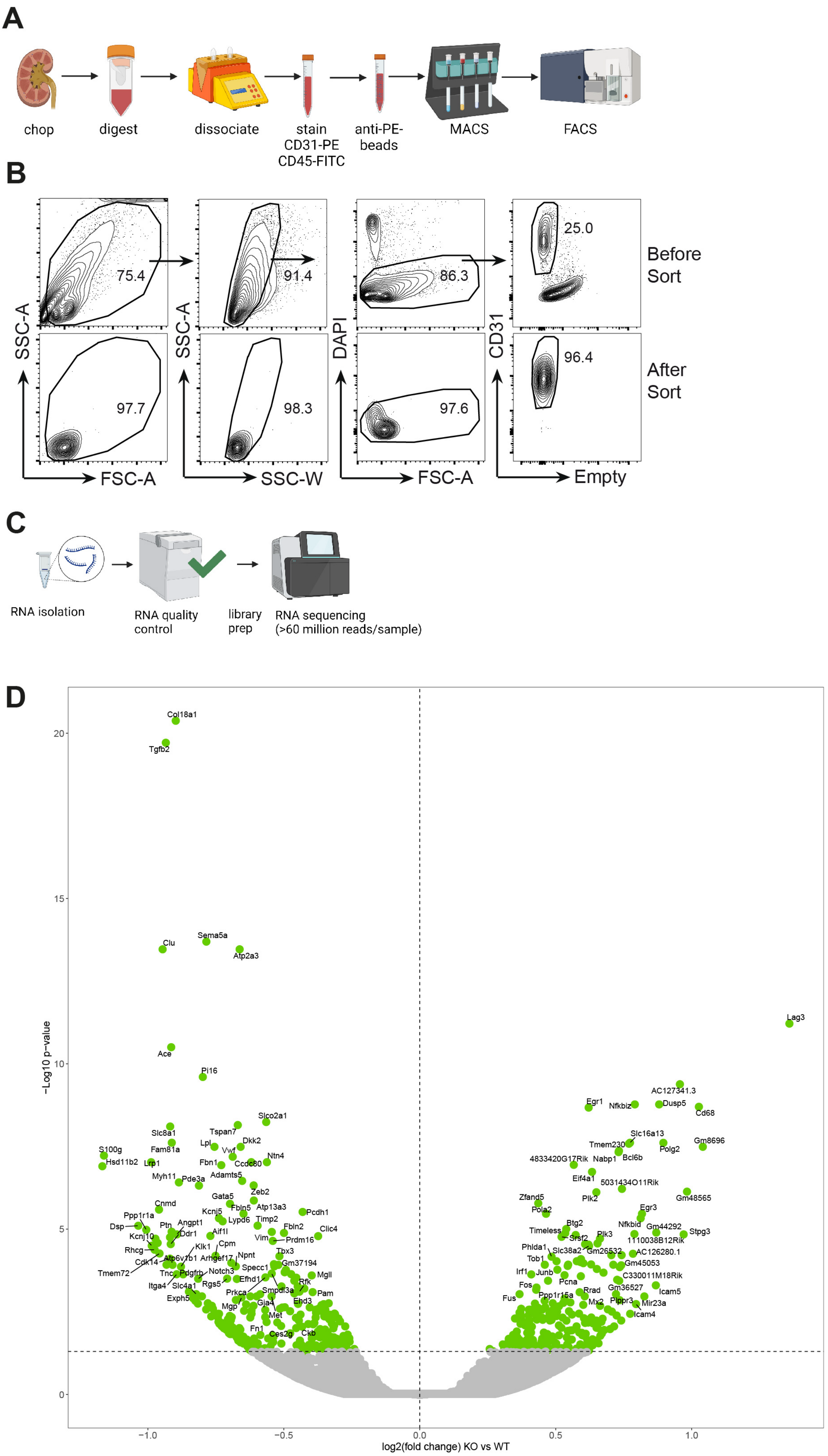
A
chop
digest
dissociate
stain
CD31-PE
CD45-FITC
anti-PE-
beads
MACS
FACS
B
75.4
91.4
86.3
25.0
Before
Sort
97.7
98.3
97.6
96.4
After
Sort
SSC-A
SSC-A
DAPI
CD31
FSC-A
SSC-W
FSC-A
Empty
C
RNA isolation
RNA quality
control
library
prep
RNA sequencing
(>60 million reads/sample)
D
Col18a1
Tgfb2
Sema5a
Clu
Atp2a3
Ace
Pi16
Lag3
AC127341.3
Egr1
Nfkbiz
Dusp5
Cd68
Slc16a13
Tmem230
Polg2
Gm8696
4833420G17Rik
Nabp1
Bcl6b
Eif4a1
5031434O11Rik
Zfand5
Plk2
Gm48565
Pola2
Egr3
Timeless
Btg2
Nfkbid
Gm44292
Stpg3
Srsf2
Plk3
1110038B12Rik
Phlda1
Slc38a2
Gm26532
AC126280.1
Tob1
Gm45053
Irf1
Junb
C330011M18Rik
Fos
Pcna
Rrad
Gm36527
Icam5
Fus
Ppp1r15a
Mx2
Pippr3
Mir23a
Icam4
Slco2a1
Slc8a1
Tspan7
Dkk2
S100g
Fam81a
Lpl
Vwf
Ntn4
Hsd11b2
Lrp1
Fbn1
Ccdc80
Myh11
Pde3a
Adamts5
Zeb2
Gata5
Cnmd
Kcnj5
Fbln5
Atp13a3
Pcdh1
Ppp1r1a
Lypd6
Timp2
Dsp
Ptn
Angpt1
Fbln2
Clic4
Kcnj10
Ddr1
Aif1l
Vim
Prdm16
Rhcg
Klk1
Cpm
Tbx3
Cdk14
Atp6v1b1
Arhgef17
Npnt
Gm37194
Tmem72
Tnc
Pdgfrb
Notch3
Specc1
Mgll
Itga4
Slc4a1
Rgs5
Efhd1
Rfk
Exph5
Prkca
Smpdl3a
Pam
Mgp
Gja4
Ehd3
Met
Fn1
Ces2g
Ckb
−Log10 p−value
log2(fold change) KO vs WT
20
15
10
5
0
−1.0
−0.5
0.0
0.5
1.0

Supplementary Figure 5: RNA seq cell-isolation strategy and analysis. **A**) Sketch of EC single cell isolation and enrichment protocol, A, C created with biorender.com. **B**) $CD31^{+}$ cell purity after MACS-Sorting (upper panel, input) and after FACS-sorting (lower panel) **C**) From single EC to RNAseq: 2ng of RNA were used for library preparation with the w 'SMARTer Stranded Total RNA-Seq Kit v2 – Pico Input Mammalian' (Takara). Generated libraries were barcoded by dual indexing approach and were finally amplified with 12 cycles of PCR. **D**) Volcano plot showing in green the genes significantly up- or downregulated in *Rbpj*$^{\Delta EC}$ EC ("KO") as compared to control EC ("WT"), at a threshold of adj. p-value < 0.05 (See methods section and supplementary data file 1,4.)

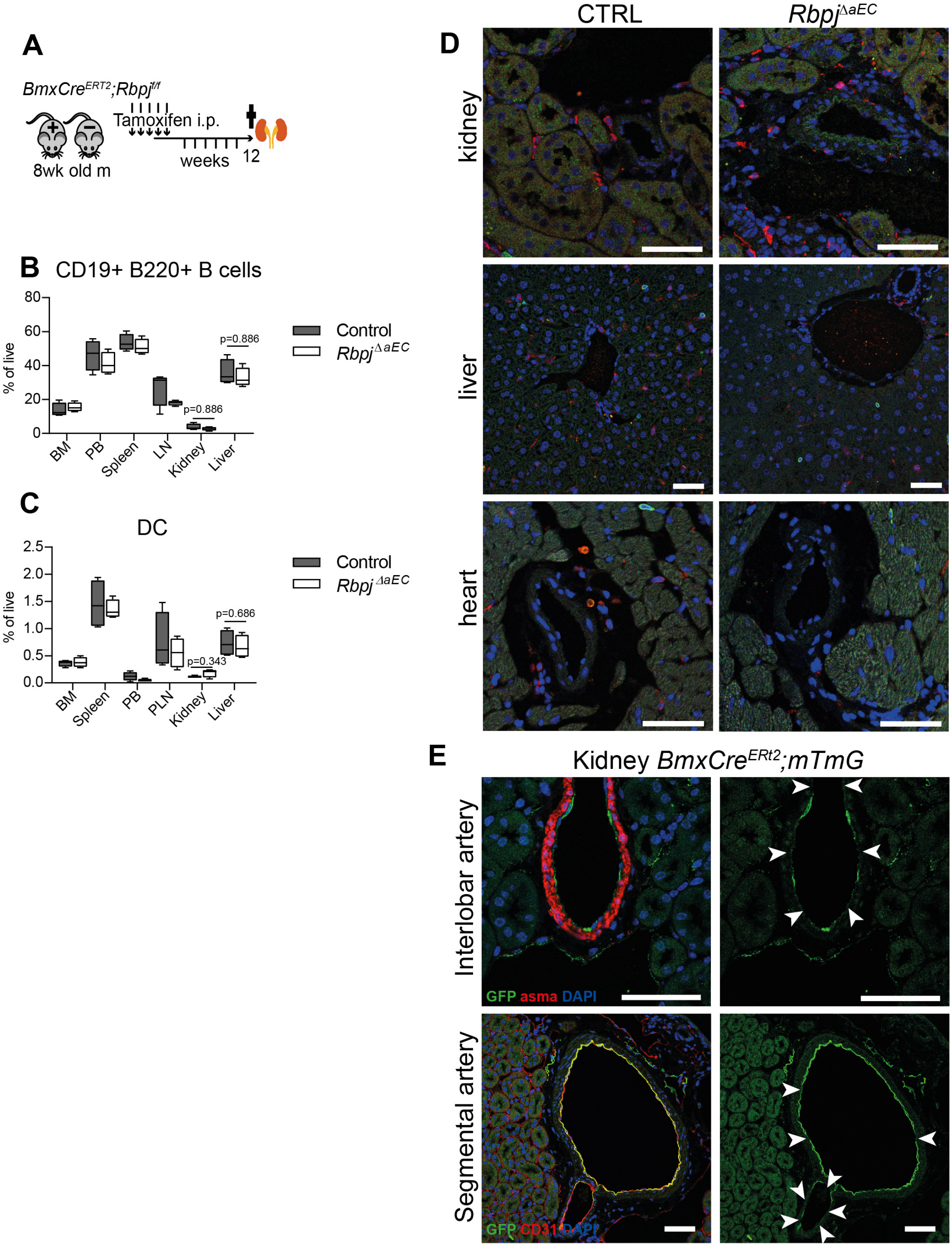

Supplementary Figure 6: Arterial EC-specific deletion of Rbpj.

A) Experimental outline analogous to Fig. 1A. **B**) Flow cytometry of B lymphocytes across bone marrow, blood, spleen, lymph node, kidney and liver from *BmxCre*^{ERT2}*;Rbpj*$^{fl/fl}$ mice and Cre-negative littermates. N=4 mice/group. **C**) Flow cytometry of dendritic cells across the same organ panel. N=4 mice/group. B and C: box plots with mean (line), 25-75% (box) and min/max (whiskers). Two-tailed Mann

Whitney test. Source data are provided with this paper. **D**) Immunofluorescence staining of B220 (green) and CD3 (red) in Ctrl and BMX-cre mediated Rbpj knockout, representative images, n= 4 mice/group. **E**) *BMX-Cre*^{ERT2}*;mTmG* recombination control induced in adult age; rehydrated paraffin sections. Several kidney sections from different areas stained from n=2 mice. Green, GFP visualized via anti-GFP-Antibody, shows Cre-mediated recombination. Other channels as indicated. White arrow heads point to non-recombined endothelial cells. All scale bars in D and E, 50µm.

Supplementary Table 1: List of antibodies used for flow cytometry.

Antibody	Clone	Label		Company	Cat. #
CD32/16	93	Unlabeled	1:200	Biolegend	101319
CD45	30-F11	AF700	1:400	Biolegend	103128
F4/80	BM8	APC	1:100	Biolegend	123116
CX3CR1	SA011F11	PE	1:200	Biolegend	149005
CD115	AFS98	AF488	1:200	Biolegend	135511
CD117	2B8	APC-Cy7	1:100	Biolegend	105803
CD19	6D5	Bio	1:400	Biolegend	115504
CD19	6D5	PE-Cy7	1:400	Biolegend	115519
B220	RA3-6B2	Bio	1:400	Biolegend	103203
B220	RA3-6B2	BV650	1:400	Biolegend	103241
CD3	17A2	Bio	1:200	Biolegend	100243
CD3	17A2	Pacific Blue	1:200	Biolegend	100213
Ter119	Ter119	Bio	1:400	Biolegend	116203
NK1.1	PK136	Bio	1:200	Biolegend	108704
NK1.1	PK136	PE	1:200	Biolegend	108707
Ly6G	1A8	Bio	1:400	Biolegend	127603
CD11b	M1/70	Pacific Blue	1:400	Biolegend	101224
Ly6C	HK1.4	PE-Cy7	1:1400	Biolegend	128018
I-A/I-E	M5/114.15.2	BV510	1:400	Biolegend	107635
CD11c	N418	BV605	1:400	Biolegend	117334
CD31	390	FITC	1:100	Serotec	MCA1364F
CD21/35	7E9	APC-Cy7	1:400	Biolegend	123417
CD23	B3B4	Pacific Blue	1:200	Biolegend	101615
IgM	RMM-1	PE	1:100	Biolegend	406507
IgD	11-26c.2a	AF647	1:400	Biolegend	405707
CD4	GK1.5	APC-Cy7	1:200	Biolegend	100413
CD8a	53-6.7	BV650	1:400	Biolegend	100741
CD43	S7	PerCP-Cy5.5	1:400	BD Pharmingen	562865
CD43	S11	PerCP-Cy5.5	1:400	Biolegend	143219
CD144	11D4.1	PE	1:100	BD Pharmingen	562243
Streptavidin		PE-Dazzle 594	1:400	Biolegend	405247

Supplementary Table 2: List and sequences of primer pairs used for QPCR.

Gene name	Forward-Primer	Reverse-Primer
Rps9	GGA TTT CTT GGA GAG GCG GC	ACC TGC TTG CGG ACC CTA AT
Havcr1 (Kim1)	ATG AAT CAG ATT CAA GTC TTC	TCT GGT TTG TGA GTC CAT GTG
Cxcl13	TGA GGC TCA GCA CAG CAA	ATG GGC TTC CAG AAT ACC G
Cxcl12	CCA AAC TGT GCC CTT CAG AT	ATT TCG GGT CAA TGC ACA CT
Cxcr4	TGG AAC CGA TCA GTG TGA GT	GGG CAG GAA GAT CCT ATT GA
Cxcr5	GTG ACC TCT CTC GGC TTC TG	AGA CTA CTC TTG CGC CAG TTG
Hey1	GCG CGG ACG AGA ATG GAA AC	GGC GCT TCT CGA TGA TGC CT
Dll1	TCC GAT TCC CCT TCG GCT TC	TGG GTT TTC TGT TGC GAG GT
Dll4	GGC CGG GAA CCT TCT CAC TC	TTT CCT GGC GAA GTC TCT GGC
Il7	TCT GCT GCC TGT CAC ATC ATC	GGA CAT TGA ATT CTT CAC TGA TAT TCA
Jagged1	CAA ATG AGT GCG AGG CCA AAC CTT	AGC CAG GAA GGC AAT CAC AGT AGT
Notch1	AGT GTC AGA GGC CAG CAA GAA GAA	TGA TTG TCG TCC ATC AGA GCA CCA
EphrinB2	TTC TGC TGG ATC AGC CAG GAA TCA	ACC TGG ATT TGG CTT CAC AAA GGG
Vcam1	TCT TAC CTG TGC GCT GTG AC	ACT GGA TCT TCA GGG AAT GAG T
Madcam	TGT CAG ACA CAG GCA CTC CT	AAG GAA CTC CGG GGA CAC
Sell	CCA TGG AAC TCA CTG TTG GA	AAA TCT GTG TAA TTT TGC TTG CAG
Selp	AAT GCC CCT TGA ACC CTC AC	GAC CGG GTT TCT TAA GGG GT
Rankl	GGA TGA AAC AAG CCT TTC AGG	ACA TCC AAC CAT GAG CCT TC
Ccl21	TCC AAG GGC TGC AAG AGA	TGA AGT TCG TGG GGG ATC T
Baff	CAG GGA CCA GAG GAA ACA GA	TTT CTG AGG TTC ATT CCA TTA TCA
Aplnr	GTG CTC TGG ACC GTG TTT C	CAC CAC AAA GGT CAA GTC AGC
Nppa	GCC GGT AGA AGA TGA GGT CA	GGG CTC CAA TCC TGT CAA TC
