# Supplementary material for: Loss of vascular endothelial notch signaling promotes spontaneous formation of tertiary lymphoid structures
Source: Nat Commun. 2022 Apr 19;13:2022. doi: 10.1038/s41467-022-29701-x (PMC9018798; doi:10.1038/s41467-022-29701-x)
Supplement: Supplementary file 3 — Description of Additional Supplementary Files [file 41467_2022_29701_MOESM3_ESM.pdf]

Description of Additional Supplementary files

File name: Supplementary Data 1
Description: Differential gene expression analysis results obtained by using DESeq2 after RNA sequencing of endothelial cells sorted from kidneys of KO (loss-of-function of Rbpj) mice or control (CTRL) mice. Results were not calculated for genes that were not detected at all (0 counts). (Excel Table for download.)

File name: Supplementary Data 2
Description: GSEA results obtained with clusterProfiler of the Gene Ontology terms (with function gseGO, see Methods). For GSEA, the Wald statistics of the genes with mean log2(normalized counts) above 3.23 were used. (Excel Table for download.)

File name: Supplementary Data 3
Description: NES values of GO terms selected for Fig. 5B. (Excel Table for download.)

File name: Supplementary Data 4
Description: Heatmap of EC RNAseq significantly changed genes between groups. (High resolution pdf for download.)

File name: Supplementary Movie 1
Description: Wholemount kidney staining and light sheet imaging of CD31 (red) and B220 (green) of Rbpj$\Delta$EC kidney as in Fig. 2E; 3D reconstruction and movie with IMARIS software. (see file for download.)

File name: Supplementary Movie 2
Description: Wholemount kidney staining and light sheet imaging of Prox1/Lyve1 (purple) and B220 (green) of Rbpj$\Delta$EC kidney as in Fig. 3E; 3D reconstruction and movie with IMARIS software. (see file for download.)
