# Supplementary figures and images for: Loss of vascular endothelial notch signaling promotes spontaneous formation of tertiary lymphoid structures
Source: Nat Commun. 2022 Apr 19;13:2022. doi: 10.1038/s41467-022-29701-x (PMC9018798; doi:10.1038/s41467-022-29701-x)

Differentially expressed genes

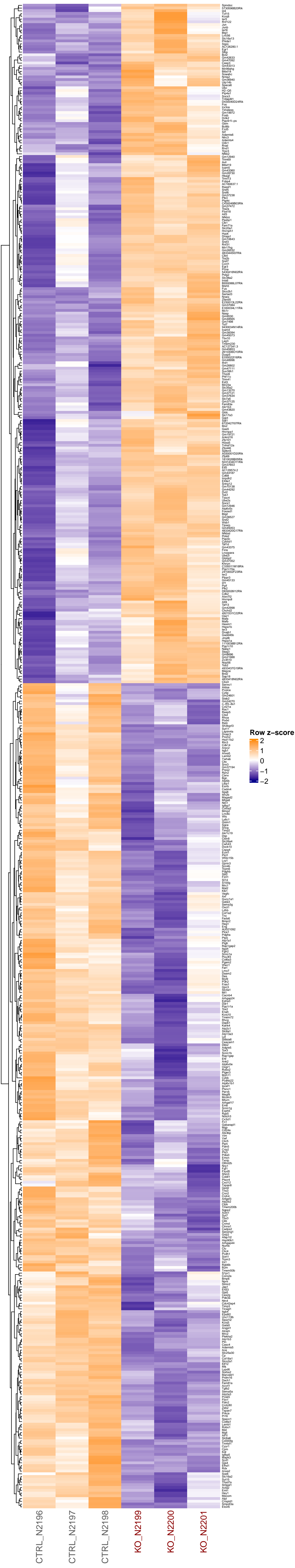

Supplement: Supplementary file 7 — Supplementary Data 4 [file 41467_2022_29701_MOESM7_ESM.pdf]
