# Supplementary material for: Loss of vascular endothelial notch signaling promotes spontaneous formation of tertiary lymphoid structures
Source: Nat Commun. 2022 Apr 19;13:2022. doi: 10.1038/s41467-022-29701-x (PMC9018798; doi:10.1038/s41467-022-29701-x)
Supplement: Supplementary file 10 — Reporting Summary [file 41467_2022_29701_MOESM10_ESM.pdf]

nature portfolio

Corresponding author(s): Florian P. Limbourg

Last updated by author(s): Feb 24, 2022

Reporting Summary

Nature Portfolio wishes to improve the reproducibility of the work that we publish. This form provides structure for consistency and transparency in reporting. For further information on Nature Portfolio policies, see our Editorial Policies and the Editorial Policy Checklist.

Statistics

For all statistical analyses, confirm that the following items are present in the figure legend, table legend, main text, or Methods section.

n/a	Confirmed	
☐	☒	The exact sample size (*n*) for each experimental group/condition, given as a discrete number and unit of measurement
☐	☒	A statement on whether measurements were taken from distinct samples or whether the same sample was measured repeatedly
☐	☒	The statistical test(s) used AND whether they are one- or two-sided<br>*Only common tests should be described solely by name; describe more complex techniques in the Methods section.*
☒	☐	A description of all covariates tested
☐	☒	A description of any assumptions or corrections, such as tests of normality and adjustment for multiple comparisons
☐	☒	A full description of the statistical parameters including central tendency (e.g. means) or other basic estimates (e.g. regression coefficient) AND variation (e.g. standard deviation) or associated estimates of uncertainty (e.g. confidence intervals)
☒	☐	For null hypothesis testing, the test statistic (e.g. *F*, *t*, *r*) with confidence intervals, effect sizes, degrees of freedom and *P* value noted<br>*Give P values as exact values whenever suitable.*
☒	☐	For Bayesian analysis, information on the choice of priors and Markov chain Monte Carlo settings
☒	☐	For hierarchical and complex designs, identification of the appropriate level for tests and full reporting of outcomes
☒	☐	Estimates of effect sizes (e.g. Cohen's *d*, Pearson's *r*), indicating how they were calculated

Our web collection on statistics for biologists contains articles on many of the points above.

Software and code

Policy information about availability of computer code

Data collection	FlowJo Software (Version v10.8.1, Treestar); LightCycler96 Version 1.1.0.1320 (© 2011 Roche Diagnostics International)<br>BD FACSDiva Software v8.0.1; LASX (Leica); ZenBlue (Zeiss)
Data analysis	FlowJo Software (Version v10.8.1, Treestar); MS Excel; GraphPad Prism Version 7 (SF) and 9 (TK); FIJI / ImageJ2; Imaris Software 7.6.5 Version I (Bitplane/Oxford Instruments)<br>bcl2fastq Conversion Software version v2.20.0.422 (Illumina); nfcore/rnaseq (version 1.4.2); DESeq2 (Galaxy Tool Version 2.11.40.2); Qlucore Omics explorer ; "gseGO" function of the clusterProfiler package (v. 4.0.5 79) in R (v. 4.0.1); Nextflow

For manuscripts utilizing custom algorithms or software that are central to the research but not yet described in published literature, software must be made available to editors and reviewers. We strongly encourage code deposition in a community repository (e.g. GitHub). See the Nature Portfolio guidelines for submitting code & software for further information.

Data

Policy information about availability of data

All manuscripts must include a data availability statement. This statement should provide the following information, where applicable:

- Accession codes, unique identifiers, or web links for publicly available datasets
- A description of any restrictions on data availability
- For clinical datasets or third party data, please ensure that the statement adheres to our policy

The RNA sequencing data generated in this study, including raw sequencing files and a table of preprocessed counts per gene per sample, are publicly available, and have been deposited in the NCBI's Gene Expression Omnibus under accession number GSE193544 [https://www.ncbi.nlm.nih.gov/geo/query/acc.cgi?

acc=GSE193544].
We used public data sets from the European cDNA bank cohort, the Nephrotic Syndrome Study Network, and the Vasculitis Clinical Research Consortium51 obtained at NCBI (GSE104948, (https://www.ncbi.nlm.nih.gov/geo/query/acc.cgi?acc=GSE104948) and GSE104954 (https://www.ncbi.nlm.nih.gov/geo/query/acc.cgi?acc=GSE104954).

Field-specific reporting

Please select the one below that is the best fit for your research. If you are not sure, read the appropriate sections before making your selection.

[x] Life sciences   [ ] Behavioural & social sciences   [ ] Ecological, evolutionary & environmental sciences

For a reference copy of the document with all sections, see nature.com/documents/nr-reporting-summary-flat.pdf

Life sciences study design

All studies must disclose on these points even when the disclosure is negative.

Sample size	For all experiments in this study, biometrical planning involved power analysis to determine Sample size. We used G*Power software (Faul, F., Erdfelder, E., Buchner, A., & Lang, A.-G. (2009). Statistical power analyses using G*Power 3.1: Tests for correlation and regression analyses. Behavior Research Methods, 41, 1149-1160.)
Data exclusions	Data from animals with unclear gentype or recombination were excluded
Replication	Data were reproduced by replicates from at least 3 indepdendent litters or runs
Randomization	Genotyping defined if the mice were Control (= cre negative) or experimental (= cre positive).<br>For the IRI-experiment, mice were randomly assigned SHAM or IRI group.
Blinding	During data collection and processing, investigators were blinded to group allocation.

Reporting for specific materials, systems and methods

We require information from authors about some types of materials, experimental systems and methods used in many studies. Here, indicate whether each material, system or method listed is relevant to your study. If you are not sure if a list item applies to your research, read the appropriate section before selecting a response.

Materials & experimental systems

n/a	Involved in the study	
[ ]	[x]	Antibodies
[x]	[ ]	Eukaryotic cell lines
[x]	[ ]	Palaeontology and archaeology
[ ]	[x]	Animals and other organisms
[x]	[ ]	Human research participants
[x]	[ ]	Clinical data
[x]	[ ]	Dual use research of concern

Methods

n/a	Involved in the study	
[x]	[ ]	ChIP-seq
[ ]	[x]	Flow cytometry
[x]	[ ]	MRI-based neuroimaging

Antibodies

Antibodies used

For Flow cytometry:
Antibody Clone Label Dilution used Company Cat. #
CD32/16 93 Unlabeled 1:200 Biolegend 101319
CD45 30-F11 AF700 1:400 Biolegend 103128
F4/80 BM8 APC 1:100 Biolegend 123116
CX3CR1 SA011F11 PE 1:200 Biolegend 149005
CD115 AFS98 AF488 1:200 Biolegend 135511
CD117 2B8 APC-Cy7 1:100 Biolegend 105803
CD19 6D5 Bio 1:400 Biolegend 115504
CD19 6D5 PE-Cy7 1:400 Biolegend 115519
B220 RA3-6B2 Bio 1:400 Biolegend 103203
B220 RA3-6B2 BV650 1:400 Biolegend 103241
CD3 17A2 Bio 1:200 Biolegend 100243
CD3 17A2 Pacific Blue 1:200 Biolegend 100213
Ter119 Ter119 Bio 1:400 Biolegend 116203
NK1.1 PK136 Bio 1:200 Biolegend 108704

	NK1.1 PK136 PE 1:200 Biolegend 108707<br>Ly6G 1A8 Bio 1:400 Biolegend 127603<br>CD11b M1/70 Pacific Blue 1:400 Biolegend 101224<br>Ly6C HK1.4 PE-Cy7 1:1400 Biolegend 128018<br>I-A/I-E M5/114.15.2 BV510 1:400 Biolegend 107635<br>CD11c N418 BV605 1:400 Biolegend 117334<br>CD31 390 FITC 1:100 Serotec MCA1364F<br>CD21/35 7E9 APC-Cy7 1:400 Biolegend 123417<br>CD23 B3B4 Pacific Blue 1:200 Biolegend 101615<br>IgM RMM-1 PE 1:100 Biolegend 406507<br>IgD 11-26c.2a AF647 1:400 Biolegend 405707<br>CD4 GK1.5 APC-Cy7 1:200 Biolegend 100413<br>CD8a 53-6.7 BV650 1:400 Biolegend 100741<br>CD43 S7 PerCP-Cy5.5 1:400 BD Pharmingen 562865<br>CD43 S11 PerCP-Cy5.5 1:400 Biolegend 143219<br>CD144 11D4.1 PE 1:100 BD Pharmingen 562243<br>Streptavidin PE-Dazzle 594 1:400 Biolegend 405247<br><br>For Immunofluorescence staining:<br>PDPN, Clone RTD4E10, abcam ab11936, dilution 1:400; Lyve1, R&D, BAF2125, dilution 1:100; B220, Clone RA3-6B2, Life Technologies 14-0452-82, dilution 1:100; CD3, DAKO A0452, dilution 1:100; CXCL13, R&D, AF470, dilution 1:1000 ; KI67, Thermo Fisher 14-5698-82, dilution 1:100; NG2, Millipore #AB5320, dilution 1:100; ER-TR7, BMA, dilution 1:100; Rankl, Clone IK22/5, eBioscience 14-5952-81, dilution 1:100; PNAd, Clone MECA79, Biolegend, dilution 1:100; Erg, Clone EPR3864, Abcam ab92513, dilution 1:400, Prox1, R&D, AF2727, dilution 1:100; CD31, Clone 390, Biolegend 122407, dilution 1:100; anti-GFP, Aves GFP-1010, dilution 1:500; Anti-asma-AF488, Clone 1A4, BD biosciences, dilution 1:300; asma-Cy3, Clone 1A4, Sigma C6198, dilution 1:300; GL7-FITC, Biolegend 144603, dilution 1:100; IgD-AF647, Clone 11-26c.2a, Biolegend #405707, dilution 1:100; CD21/35-FITC, Clone 7G6, BD Pharmingen 561769, dilution 1:100; anti-Prox1 (R&D AF2727, 1:100) and Anti-LYVE1 (R&D BAF2125, 1:100
Validation	For flow cytometry (see upper table), all antibodies were tested in flow cytometry for specificity of staining and titrated for optimal dilution before we used them experimentally.<br>For histology (see lower list), all antibodies were commercially available, tested and validated; in our laboratory, we additionally established staining on control slides of mouse organs that should express the antigen (immunofluorescence staining and antibody dilution titration on control slides). For all stainings, we used positive control slides (=tissue that should express the antigen) and did negative controls (no primary ab / no secondary ab).

Animals and other organisms

Policy information about studies involving animals; ARRIVE guidelines recommended for reporting animal research

Laboratory animals	The following genetic mouse strains were used (male mice, age 7-9 weeks at start of experiment) unless otherwise specified:<br>B6-Tg(Cdh5(PAC)-cre/ERT2)1Rha Rbpsuh tm3Hon/Rbrc; (=Vecad;Rbpj)<br>B6-Tg(Bmx(PAC)-cre/ERT2)1Rha Rbpsuh tm3Hon/Rbrc; (=BMX;Rbpj)<br>B6-Tg(Prox1-Cre/ERT2)1Rha Rbpsuh tm3Hon/Rbrc; (=Prox1;Rbpj)<br>B6- Gt(ROSA)26Sortm4(ACTB-tdTomato,-EGFP)Luo/J Tg(Prox1-Cre/ERT2) (=Prox1;mTmG)<br>B6-TGt(ROSA)26Sortm4(ACTB-tdTomato,-EGFP)Luo/J g(Bmx(PAC)-cre/ERT2) (=BMX;mTmG)<br>B6-Gt(ROSA)26Sortm9(CAG-tdTomato)Hze Tg(Cdh5-cre/ERT2)1Rha (=Vecad;TdTomato)<br>aMHC-Cre; STAT3flox/flox (as published in Hilfiker-Kleiner D et al, 2004) - male mice at age 6-8 months<br>LDLr-/- mice purchased from Jackson Laboratories (strain 002207)
Wild animals	No wild animals were used in this study.
Field-collected samples	No field collected samples were used in this study.
Ethics oversight	All animal studies were undertaken in accordance with German Animal Welfare legislation and with the European Communities Council Directive 2010/63/EU for the protection of animals used for experimental purposes. All experiments were approved by the Local Institutional Animal Care and Research Advisory Committee and permitted by the relevant local authority for animal protection.<br>Experiments conducted at Hannover Medical School had approval of the local animal welfare board (LAVES, Lower Saxony, Animal Studies Committee), experiments conducted at Lausanne University were performed with the approval of the Animal Ethics Committee of Vaud, Switzerland.

Note that full information on the approval of the study protocol must also be provided in the manuscript.

Flow Cytometry

Plots

Confirm that:

- [x] The axis labels state the marker and fluorochrome used (e.g. CD4-FITC).
- [x] The axis scales are clearly visible. Include numbers along axes only for bottom left plot of group (a 'group' is an analysis of identical markers).
- [x] All plots are contour plots with outliers or pseudocolor plots.
- [x] A numerical value for number of cells or percentage (with statistics) is provided.

Methodology

Sample preparation	Mice were sacrificed and blood was collected from the Vena cava in Na2EDTA containing tubes; spleens, kidneys, bones and para-aortic lymph nodes were excised and kept on ice during preparation. Spleens and lymph nodes were pressed, resuspended in PBS (Millipore) and filtered through a 70µm mesh; blood and bone marrow from tibia and femur were filtered as above. Erythrocytes were removed from splenic and blood cell suspensions by red blood cell lysis buffer (Biolegend). Kidneys were minced to small pieces (<1mm), then digested with Collagenase II (Worthington) 500U/ml for 2x 21min at 37°C, interrupted by 1-2 courses of tissue dissociation (GentleMACS, program B). Cells were washed with PBS and filtered through a 70µm mesh several times before proceeding with staining. For CD23 staining experiments, kidneys were pressed and filtered as indicated above and lymphocytes were isolated using double (70%-40%) percoll (GE Healthcare) density gradient centrifugation. After extensive washing cells from all organs were resuspended into PBS, counted using a Countess II automatic cell counter (Thermofisher Scientifics) and used for flow cytometry.
Instrument	LSR II flow cytometer (BD Biosciences)
Software	FlowJo software (TreeStar) v10.8.1
Cell population abundance	For EC single cell sort, total yield of EC (CD31+CD45- cells) was about 5x10^5 to 1x10^6 live cells per kidney.
Gating strategy	For Gating strategy please see supplementary figure 1,2,6.

- [x] Tick this box to confirm that a figure exemplifying the gating strategy is provided in the Supplementary Information.
